# PPAR-gamma induced AKT3 expression increases levels of mitochondrial biogenesis driving prostate cancer

**DOI:** 10.1038/s41388-021-01707-7

**Published:** 2021-03-02

**Authors:** Laura C. A. Galbraith, Ernest Mui, Colin Nixon, Ann Hedley, David Strachan, Gillian MacKay, David Sumpton, Owen J. Sansom, Hing Y. Leung, Imran Ahmad

**Affiliations:** 1 Cancer Research UK Beatson Institute, Switchback Road, Bearsden, Glasgow, G61 1BD UK; 2 Institute of Cancer Sciences, University of Glasgow, Bearsden, Glasgow, G61 1QH UK

**Keywords:** Cancer models, Prostate cancer

## Abstract

Peroxisome Proliferator-Activated Receptor Gamma (PPARG) is one of the three members of the PPAR family of transcription factors. Besides its roles in adipocyte differentiation and lipid metabolism, we recently demonstrated an association between PPARG and metastasis in prostate cancer. In this study a functional effect of PPARG on AKT serine/threonine kinase 3 (AKT3), which ultimately results in a more aggressive disease phenotype was identified. AKT3 has previously been shown to regulate PPARG co-activator 1 alpha (PGC1α) localisation and function through its action on chromosome maintenance region 1 (CRM1). AKT3 promotes PGC1α localisation to the nucleus through its inhibitory effects on CRM1, a known nuclear export protein. Collectively our results demonstrate how PPARG over-expression drives an increase in AKT3 levels, which in turn has the downstream effect of increasing PGC1α localisation within the nucleus, driving mitochondrial biogenesis. Furthermore, this increase in mitochondrial mass provides higher energetic output in the form of elevated ATP levels which may fuel the progression of the tumour cell through epithelial to mesenchymal transition (EMT) and ultimately metastasis.

## Introduction

Prostate cancer (PC) is the most common cancer in adult males in the developed world and the second leading cause of cancer deaths [1]. It has long been known that PC progression is dependent on androgens [2] and as such many treatments centre around inhibition of androgens and the androgen receptor. However, some PCs develop resistance to these treatments, developing a “castrate resistant” state, which ultimately results in metastasis and death [3]. It is this cohort of patients that have the greatest unmet need, and for which new therapies are desperately sought.

In a transposon-based Sleeping- Beauty genetic screen [4], *PPARG* was identified as being expressed at elevated levels in advanced, metastatic PC. Further analysis of prostate tumour lysates revealed that up-regulation of PPARG correlated with increased levels of its downstream metabolic effectors such as Fatty acid synthase (FASN), acetyl-CoA carboxylase (ACC) and ATP citrate lyase (*ACLY*) [4, 5]. Phenotypically this was associated with increased lung and lymph-nodes metastasis, suggesting a way of stratifying patients with PPARG or FASN activation to targeted treatments for their aggressive disease. In this study we further investigate the mechanism by which PPARG drives aggressive PC by identifying a link between PPARG and elevated mitochondrial biogenesis through its effects on AKT3, and PGC1α.

PGC1α is a well-known regulator of mitochondrial biogenesis [6] and is highly expressed in tissues where energy demand is high [7, 8]. As its name suggests PGC1α is a co-factor for PPARG [8], and as well as being the master regulator for mitochondrial biogenesis, also affects the transcription of key genes required for oxidative metabolism [9], consequently affecting mitochondrial function and ATP output.

AKT3 is one of three isoforms of AKT (AKT1 & AKT2 being the other isoforms) that regulate a number of diverse cellular processes including metabolism, proliferation, cell survival, growth and angiogenesis [10]. Each isoform performs a number of distinct functions that cannot be compensated for by another [11, 12]. AKT3 has been shown to affect mitochondrial mass and function [13, 14], by influencing the localisation of PGC1α [14], which is fundamental for its role in mitochondrial biogenesis [15]. Mechanistically, AKT3 affects the stability of CRM1, the major nuclear export protein, which exports PGC1α from the nucleus to the cytoplasm [15].

In this study we identify a previously unknown role for PPARG in up-regulating AKT3, which promotes PGC1α localisation to the nucleus and consequently increases mitochondrial mass and function, increasing ATP levels, tumour growth and metastasis.

## Methods

### Orthograft prostate cancer model

For prostate orthograft animal experiments, CD1-nude male mice were obtained from Charles River Research Models & Services (UK) at 6–8 weeks of age and cells injected into the anterior prostate lobes. Further details given in supplementary methods.

### RNA-seq

Quality of the purified RNA was tested on an Agilent 2200 Tapestation using RNA screentape. Libraries for cluster generation and DNA sequencing were prepared following an adapted method from Fisher et al. 2011 [16] using Illumina TruSeq Stranded mRNA LT Kit. Quality and quantity of the DNA libraries was assessed on an Agilent 2200 Tapestation (D1000 screentape) and Qubit (Thermo Fisher Scientific) respectively. The libraries were run on the Illumina Next Seq 500 using the High Output 75 cycles kit (2x36cycles, paired end reads, single index). Quality checks on the raw RNASeq data files were done using fastqc version 0.10.1 [17] and fastq_screen version 0.4.2 [18]. RNASeq reads were aligned to the GRCh38 [19] version of the human genome using tophat2 version 2.0.13 [20] with Bowtie version 2.4.4.0 [21]. Expression levels were determined and statistically analysed by a combination of HTSeq version 0.9.1 [22], the R 3.3.3 environment, utilising packages from the Bioconductor data analysis suite and differential gene expression analysis based on the negative binomial distribution using the DESeq2 [23].

### Statistical analysis

Statistical analyses, with the exception of that described for the RNA-seq data, was undertaken using GraphPad Prism v8.1.1(224). The tests used comprised of un-paired two tailed *t*-tests, Mann–Whitney, ratio *t*-test, Spearman correlation and Two and One-way Anova, with post tests for multiple comparisons (detailed in figure legends). All experiments (with the exception of those involving mice) were performed in biological replicates, with technical replicates for each experiment given in the methods. For mouse experiments six mice were used for each cell line derived clone. For technical reasons only five mice were available at endpoint for mice from the KO1 and KO2 cohorts.

## Results

### PPARG is required for in vivo tumour growth

**Fig. 1:**
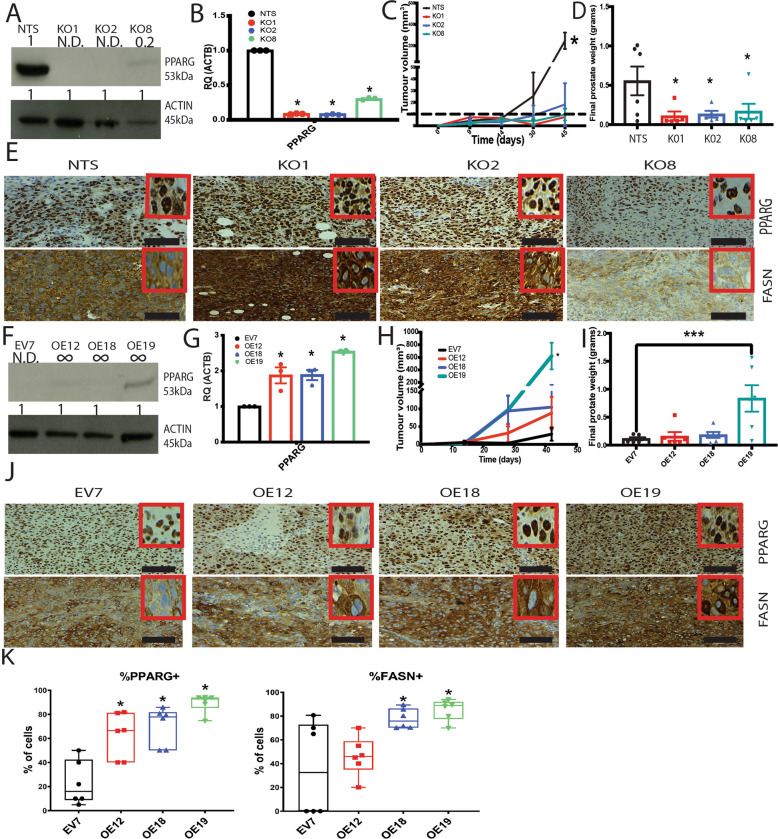
PPARG expression is necessary for tumour development in vivo. **A** Immunoblot of PC3-M CRISPR knockdown of PPARG clones (KO1, 2 & 8) and Scrambled control (Scrm), representative image of three experiments. Values above each band indicate densitometry value of band as normalised to Actin loading control and compared to first band (NTS), N.D not detected. **B** qPCR analysis for PPARG levels in clones KO1, 2 & 8, and control Scrm, three independent experiments and three technical replicates per experiment, error bars show SEM. **C** Ultrasound measurement of tumour size (KO clones & Scrm control) over time in days, ‘*’ denotes statistical significance *p* ≤ 0.05 as determined by 2-way Anova and Dunnett’s multiple comparison test. **D** Final tumour weight (KO clones & Scrm control) upon necropsy, ‘*’ denotes statistical significance *p* ≤ 0.05 as determined by one-way Anova and Holm-Sidaks multiple comparison test. For both **C** and **D** graphs represent 23 mice, at least 5 mice per cell line; ultrasound performed every two weeks until endpoint. All mice taken at the same time when the first mouse reached endpoint. **E** IHC images of PPARG KO derived orthograft tumours. Representative images from PPARG KO tumours stained for PPARG and FASN, scale bar shows 100 μm. **F** Immunoblot of DU145 PPARG over-expressing clones (OE12, 18 & 19) and control (EV7), representative image of three independent experiments. Values above each band indicate densitometry value of band as normalised to Actin loading control and compared to first band (EV7), N.D not detected. **G** qPCR analysis for PPARG levels in OE12, 18 & 19 and control EV7 qPCR three independent experiments and three technical replicates per experiment, error bars show SEM. **H** Ultrasound measurement of tumour size over days in OE clones & EV7 control, statistical significance is denoted by * where *p* ≤ 0.05 as determined by 2-way Anova and Dunnetts multiple comparison test. **I** Final tumour weight upon necropsy in OE clones & EV7 control. Statistical significance is denoted by * where *p* ≤ 0.05 determined by one-way Anova and Holm-Sidak’s multiple comparisons test. For **H** and **I** graphs represent 24 mice, 6 mice per cell line, ultrasound performed every two weeks until endpoint. All mice were taken at the same time when the first mouse reached endpoint. **J** IHC images of PPARG OE derived orthograft tumours. Representative images from tumours stained for PPARG and FASN, scale bar shows 100 μm. **K** IHC image analysis for PPARG and FASN. Graph displays average percentage positive cells over at least three samples per clone with error bars giving SEM. Statistical significance, where found, denoted by * with *p* ≤ 0.05 determined by Mann–Whitney.

Using the established PC cell lines DU145 and PC3-M, stable cell lines exhibiting over-expression or knockout of *PPARG* were generated. In PC3-M cells, which express high endogenous levels of PPARG [4], knockdown of *PPARG* was achieved using CRISPR/CAS9 technology. Three clones were selected, two with almost complete knockdown of PPARG (KO1 and KO2) and one with partial knockdown (KO8) in addition to the non-targeting control (NTS), verified by immunoblot and qPCR (Fig. 1A, B). CD1 nude mice were orthotopically implanted with PC3-M PPARG KO clones and NTS control into the anterior prostates of male mice. Mice implanted with NTS control produced tumours that were significantly bigger than those from KO clones (Fig. 1C, D). Mice implanted with KO clones produced very small or no tumour at all. Immunohistochemistry (IHC) analysis on those KO tumours that did develop showed that all expressed PPARG (Fig. 1E and Supplementary Fig. 1A). In addition the levels of Ki67, indicating proliferating cells, and downstream effector of PPARG, FASN [24], were also similar to NTS control in these tumours, with FASN expression levels correlating with PPARG (Supplementary Fig. 1B–D). The PPARG CRISPR construct used to generate the clones also contained the gene for red fluorescent protein (RFP), thus IHC for RFP could be used to determine if the tumours that had developed had derived from cells still expressing this construct. None of the tumours derived from KO1, 2 and 8 implanted cells expressed RFP (Supplementary Fig. 1E). Furthermore RNA-scope in situ hybridisation for human peptidyl Isomerase B (HuPPIB), demonstrated detectable levels in the NTS and all K0 tumours (Supplementary Fig. 1F, G), suggesting that in the tumours arose from a small sub-population of human cells that were not knocked down for PPARG. This would also account for the reduced size of the tumours in the KO1, 2 and 8 at endpoint as compared to control (NTS), as they would have smaller starting population at point of injection.

DU145 cells, which express low endogenous levels of PPARG [4], were transfected with a plasmid construct to induce constitutive PPARG over-expression (OE). Three clones were chosen, two with higher levels of PPARG as evidenced by qPCR (OE12 and OE18) and one with elevated levels of PPARG detectable by both qPCR and immunoblot (OE19), and an empty vector control (EV7) (Fig. 1F, G). CD1 nude mice were implanted with these clones of OE and EV7 control. Five out of six mice implanted with clone OE19, the clone expressing the highest level of PPARG, generated tumours. These tumours were all significantly bigger than those generated from the EV7 control (Fig. 1H, I). Clones OE12 and OE18, expressing intermediate levels of PPARG, produced tumours ranging in size between that of the EV7 and OE19 clones (Fig. 1H, I). IHC analysis demonstrated, elevated Ki67 expression (Supplementary Fig. 1H, I), and PPARG in all tumours derived from the OE clones as compared to EV7. FASN levels were also significantly elevated in clones OE18 and OE19 as compared to EV7 and observed to correlate with the increased PPARG levels (Fig. 1J, K and Supplementary Fig. 1J).

Together this data suggests an increase in proliferation and lipid metabolism regulated by PPARG, as previously observed [4]. There appears to be a requirement for PPARG in tumour growth and development in vivo, as no PPARG negative tumours developed, it suggests that not only is PPARG able to accelerate tumour growth and development, but it may be essential for the tumour establishment.

### Metabolic effects of PPARG in prostate cancer

FASN, a downstream effector of PPARG [24], was elevated in OE orthotopic tumour samples (Fig. 1J, K), suggesting a metabolic effect of PPARG. To further investigate this Seahorse bioscience stress test assays, measuring cellular oxygen consumption, were performed.

**Fig. 2:**
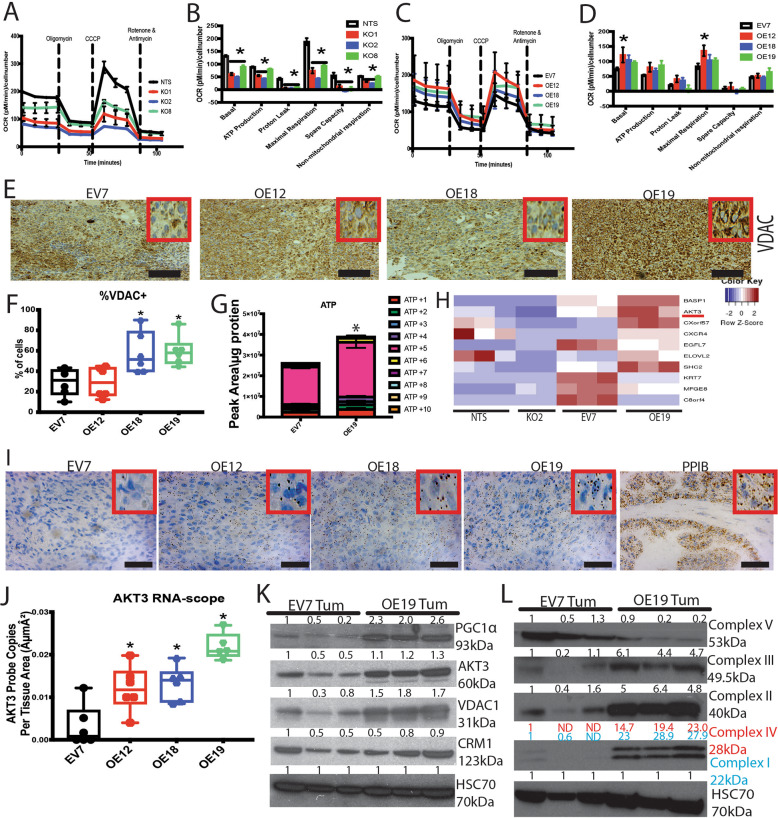
Mitchondiral effects of PPARG lead to identification of AKT3 as a novel PPARG effector gene. **A**, **B** Seahorse Bioscience mito stress test assay KO clones & Scrm control, graphs represent three independent experiments and four technical replicates per experiment, error bars show SEM. Statistical significance denoted by * where *p* ≤ 0.05 determined by 2-way Anova and Dunnetts multiple comparison test. **C**, **D** Seahorse Bioscience mito stress test assay OE clones & EV7 control, graphs represent three independent experiments and four technical replicates per experiment, error bars show SEM. Statistical significance denoted by * where *p* ≤ 0.05 by 2-way Anova and Dunnetts multiple comparison test. **E** IHC representative images for VDAC1 staining in OE & EV7 derived tumours, scale bar represents 100 μm. **F** Analysis of IHC images for VDAC1. Graph displays average percentage of positively stained cells over at least three samples with error bars giving SEM. Statistical significance, where found, denoted by * with *p* ≤ 0.05 determined by Mann–Whitney. **G** Carbon 13 labelled glucose derived ATP levels from EV7 and OE19 clones grown in 3D, unlabelled not shown. Represents three independent experiments each with three technical replicates. Bars represent average of these experiments with error bars showing SEM. Statistical significance is denoted by * where *p* ≤ 0.05 determined by 2 way Anova and Sidak’s multiple comparisons test where +5 ATP was highlighted as the significantly different isotopologue. **H** Subset of top 10 significant hits from RNA-seq analysis performed on tumour samples derived from PC3-M Scrm control, KO2, and DU-145 EV7 and OE19 clones. Three tumour samples from each cell type were used. Scrm vs. KO2 compared to EV7 vs. OE19 to identify hits that were oppositely affected in each comparison. All hits are statistically significant with *p* ≤ 0.05 and are ordered in terms of statistical significance, further details of analysis are given in the material and methods section. **I** RNA-scope in situ hybridisation representative images for AKT3 probing in OE & EV7 derived tumours, scale bar represents 50 μm. **J** Halo software analysis of RNA-scope for AKT3 on tumours derived from OE cell lines. Analysis performed on at least three prostate samples for each cell type used, graph displays average with error bars giving SEM. Statistical significance denoted with * where *p* ≤ 0.05 determined by Anova and Dunnett’s multiple comparisons test. **K** Immunoblot analysis from EV7 and OE19 derived tumour samples, three tumours per cell type. Values above each band indicate the densitometry value of the band as normalised to the loading control HSC70 and compared to the first EV7 band. Probed for PGC1a, CRM1, AKT3 and VDAC1, image representative of three independent experiments. **L** Immunoblot for the ETC complexes, complex I is labelled in blue and complex IV in red to allow the reader to identify the correct densitometry value for the corresponding bands, image representative of three independent experiments. Values above each band indicate the densitometry value of the band as normalised to the loading control HSC70 and compared to the first EV7 band.

PC3-M KO clones demonstrated a significantly reduced oxygen consumption rate (OCR), for all three KO clones as compared to NTS control, with KO1 and KO2 being significantly different to NTS in all phases of the assay (Fig. 2A, B). Furthermore, there appeared to be a “dose” dependent effect of PPARG on OCR. The partial knockdown clone KO8 displayed an OCR between that of the NTS control (on average 20% less than NTS) and complete knockouts KO1 and KO2 which had even lower OCR (on average 50% less than NTS). The DU145 PPARG over-expressing (OE) cells revealed no reciprocal advantage with increased PPARG levels (Fig. 2C, D).

A change in OCR results from differences in mitochondrial function and/or mass. Therefore, IHC was performed on the OE and EV7 derived tumours for VDAC1, a mitochondrial voltage dependant anion channel, used here as a surrogate measure for mitochondrial mass (Fig. 2E). It was observed that VDAC1 was significantly elevated in OE18 and OE19 as compared to EV7 (Fig. 2F), indicating an increase in mitochondrial mass. To determine if this increased mass was affecting metabolic output ^13^C labelled glucose metabolomics was performed on OE cells grown in 3D spheroid culture. A significant increase in ATP was observed in the OE19 clones as compared to EV7 (Fig. 2G), indicating elevated energy output concurrent with the elevated mitochondrial mass observed. To find the link between elevated mitochondrial mass, ATP production and PPARG; RNA-Seq (of orthograft samples) was undertaken (using De-Seq^2^ method, details in material and methods). From this we identified the top ten significant hits (Fig. 2H, full list given in Supplementary Fig. 2A). Within the top ten, and with a fold change of 1.8 and −7.3 for EV7 vs OE19 and NTS vs K02 respectively, *AKT3* appeared an interesting candidate given its presumptive role in mitochondrial biogenesis through CRM1 and PGC1α and metabolism [13–15, 25]. This result was validated by RNA-scope in situ hybridisation confirming statistically significant elevated *AKT3* mRNA expression in DU145 OE tumours, compared to EV7 tumours (Fig. 2I, J). Interestingly, given initial immunoblotting for PPARG (Fig. 1A, F), AKT3 levels appeared higher in OE19 as compared to NTS. When PPARG expression from in vivo experiments is compared, it does in fact appear that OE19 tumours have significantly higher PPARG expression than NTS tumours, which could account for the elevated AKT3 levels observed in OE19 tumours (Supplementary Fig. 1K).

Further validation of PPARG’s effect on this pathway was established through immunoblot of tumour lysates probing for PPARG, AKT3, PGC1α, CRM1 and VDAC1 (Fig. 2K and Supplementary Fig. 2B, C). Tumours derived from clone OE19 showed elevated AKT3, PGC1a and VDAC1 confirming the effect of PPARG on this pathway. These results were also observed for OE12 and OE18 but only in some samples and were not consistent. For OE19 most of the prostate was composed of tumour tissue, whilst for OE12 and OE18 the tumours were smaller, with only a portion of the prostate composed of tumour tissue. The rest was found to be histologically normal which may account for the variability in the protein levels shown on the immunoblot compared to IHC, where lysates derive from pulverised prostate containing both tumour and potentially normal tissue.

Tumour samples were also probed by immunoblot for each complex in the Electron Transport Chain (ETC) (Fig. 2L and Supplementary Fig. 2C). Again, in clone OE19 each of these complexes were found to be increased as compared to EV7, with the exception of Complex V. As before the results for OE12 and OE18 were mixed. These changes are concurrent with the effect of PPARG on the AKT3-CRM1-PGC1α pathway as PGC1α in addition to its role in mitochondrial biogenesis also regulates the expression of the ETC complexes [9].

Together this suggests elevated PPARG levels induce increased expression of AKT3, which, given its established functional link to PGC1α and mitochondrial biogenesis [15] would explain the increased mitochondrial mass and ATP levels observed upon PPARG over-expression. This increase in ATP levels may fuel the increase in growth and metastasis observed upon increased levels of PPARG.

### PPARG contributes to epithelial to mesenchymal transformation

**Fig. 3:**
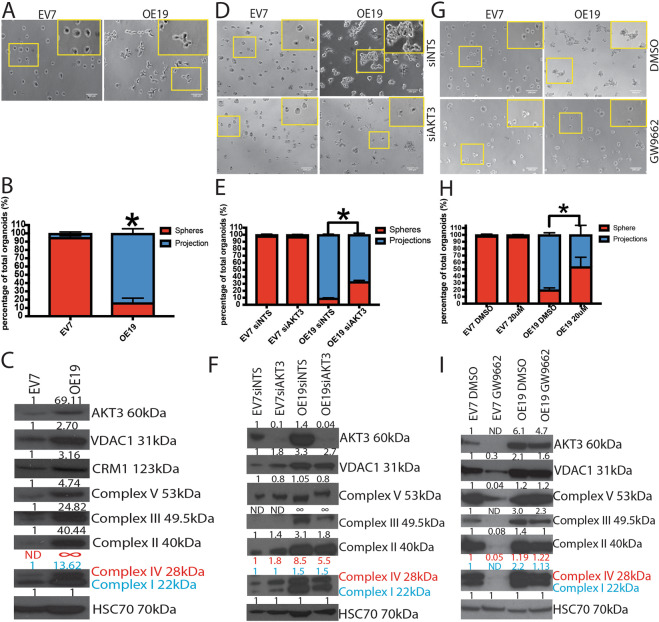
Phenotypic effects of PPARG over-expression in 3D culture regulated by the PPARG-AKT3 functional axis. **A** Characterisation of the phenotypic effect of PPARG over-expression in 3D culture representative images of EV7 and OE19 cells grown in 3D, images representative of three independent experiments. **B** Quantification of the two different spheroid appearances, represents three independent experiments and eight technical replicates per experiment. error bars show SEM Statistical significance denoted by *p ≤ 0.05 determined by 2-way Anova and Sidak’s multiple comparisons test. **C** Immunoblot analysis from 3D spheroids of EV7 and OE19 for mitochondrial ETC complexes, AKT3, CRM1 and VDAC1. HSC70 was used as loading control. Values above each band indicate the densitometry value of the band as normalised to the loading control HSC70 and compared to the EV7 band. Complex I is labelled in blue and complex IV in red to allow the reader to identify the correct densitometry value for the corresponding bands, image representative of three independent experiments. **D** siRNA knockdown of AKT3 effect in 3D culture appearance on EV7 and OE19 cells grown in 3D either treated with non-targeting siRNA (siNTS) or AKT3 targeting siRNA (siAKT3). Images representative of three independent experiments. **E** Quantification of the two different spheroid appearances following AKT3 knockdown, represents three independent experiments and ten technical replicates per experiment, error bars show SEM, Statistical significance denoted by *p ≤ 0.05 determined by 2-way Anova and Sidak’s multiple comparisons test. **F** Immunoblot analysis from lysates derived from 3D siRNA knockdown of AKT3, showing the ETC complexes, VDAC1 and AKT3. HSC70 was used for loading control. Complex I is labelled in blue and complex IV in red to allow the reader to identify the correct densitometry value for the corresponding bands, image representative of three independent experiments. Values above each band indicate the densitometry value of the band as normalised to the loading control HSC70 and compared to EV7 siNTS. **G** PPARG inhibitor effect on 3D spheroid appearance. EV7 and OE19 cells grown in 3D either treated with DMSO or PPARG inhibitor GW9662 at 20 μM. Images representative of three independent experiments. **H** Quantification of the two different spheroid appearances following GW9662 treatment, represents three independent experiments and ten technical replicates per experiment, error bars show SEM, Statistical significance denoted by *p ≤ 0.05 determined by 2-way Anova and Sidak’s multiple comparisons test. **I** Immnuoblot analysis from lysates derived from 3D spheroid treatment with PPARG inhibitor GW9662 at 20 μM, showing the ETC complexes, VDAC1 and AKT3. HSC70 was used for loading control. Complex I is labelled in blue and complex IV in red to allow the reader to identify the correct densitometry value for the corresponding bands, image representative of three independent experiments. Values above each band indicate the densitometry value of the band as normalised to the loading control HSC70 and compared to EV7 DMSO.

When culturing the KO and OE clones in 2D conditions no obvious effects of altered PPARG expression were observed, however striking differences were observed when growing them as 3D spheroids. The EV7 clones grew as uniform rounded spheroids (referred to as spheres), whilst the OE19 clones grew in a much less uniform fashion with cells protruding from the main sphere, (referred to as projections) (Fig. 3A). The percentage of sphere and projection spheroid type was quantified showing a significant difference in the number of spheres versus projections when comparing EV7 to OE19 clones (Fig. 3B). This type of spheroid morphology is suggestive of loss of cellular polarity, a key feature of epithelial to mesenchymal transition (EMT), which is proposed to be required for successful metastasis to occur [26]. Immunoblotting was performed with spheroid lysates and AKT3, VDAC1, all the complexes of the ETC, and EMT markers N-Cadherin and Vimentin were found to be elevated in OE19 as compared to EV7, (Fig. 3C and Supplementary Fig. 3A). In the OE12 and OE18 clones little or no projections were observed, however, a difference in the size of the spheres was noted. We quantified this and found that both OE12 and OE18 produced significantly bigger spheres than EV7 cells (Supplementary Fig. 3B, C). This increase in spheroid size is indicative of a growth advantage in these PPARG OE cells in 3D as compared to EV7. Immunoblotting of these spheroids showed a similar elevation in all proteins of interest in OE18 compared to EV7, however OE12 showed mixed results (Supplementary Fig. 3A, D). The less dramatic observations likely result from the expression level of PPARG in these clones as compared to OE19.

To understand the possible connection to the PPARG-AKT3 functional axis we knocked-down *AKT3* using siRNA (Fig. 3D–F). EV7 cells were unaffected whilst OE19 cells appeared to show a partial reversion back to the sphere phenotype (Fig. 3D). Upon quantification this partial reversion was found to be significant (Fig. 3E), implying the projections phenotype was affected by AKT3. Furthermore, immunoblot analysis of the lysates demonstrated the effect of AKT3 loss on mitochondrial mass as evidenced by decreased expression of VDAC1 and the ETC complexes (Fig. 3F). This indicates that in 3D AKT3 is at least partially responsible for the effects observed.

Treating the spheroids with PPARG inhibitor GW9662 or knockdown with PPARG specific siRNA, showed greater reversion than knockdown of AKT3. EV7 cells were unaffected by PPARG inhibition or knockdown, however OE19 cells showed reversion to the sphere phenotype (Fig. 3G and Supplementary Fig. 3E, F), following quantification this was shown to be statistically significant (Fig. 3H and Supplementary Fig. 3G). Immunoblot analysis of lysates derived from these treated 3D cultures revealed that as well as VDAC1 and the proteins of the ETC complexes being reduced upon PPARG inhibition so was AKT3 (Fig. 3I and Supplementary Fig. 3H).

This further demonstrates the link between AKT3 and PPARG and their downstream effects on mitochondrial mass and energy output potentially facilitating EMT progression.

### PPARG alters PGC1α localisation and function through AKT3

**Fig. 4:**
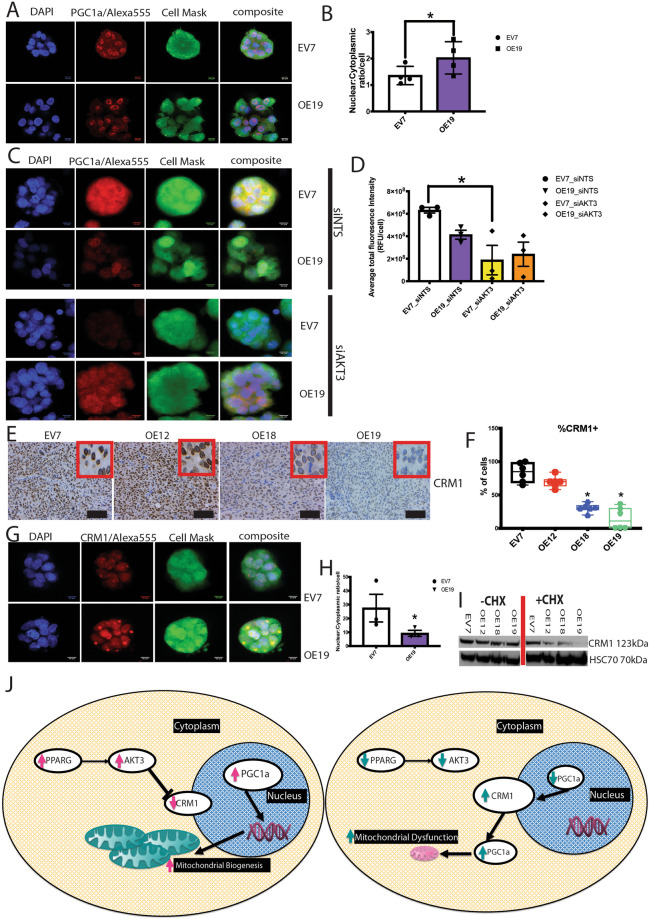
PPARG prevents PGC1α nuclear export by disrupting the stability of CRM1 through AKT3 up-regulation, and so increases mitochondrial biogenesis & function. **A** Immunofluorescence of 3D spheroids, representative images for EV7 and OE19 clones grown in 3D, fixed and stained for the nuclear stain DAPI (Blue), PGC1α/Alexa 555 (Red) and Cell volume with cell mask (green). **B** PGC1α nuclear to cytoplasmic ratio, determined through Volocity image analysis. Represents four independent experiments and six images from each experiment, bars represent average over these experiments and error bars the SEM. Statistical significance is denoted by * where *p* ≤ 0.05 determined by Mann–Whitney. **C** Immunofluorescence 3D spheroids with siRNA knockdown of AKT3, representative images for EV7 and OE19 clones grown in 3D, fixed and stained for the nuclear stain DAPI (Blue), PGC1α/Alexa 555 (Red) and Cell volume with cell mask (green). **D** Quantification of total PGC1a levels by IF following knockdown of AKT3 by siRNA in 3D culture in EV7 and OE19 clones, normalised to cell number. Three independent experiments five images per experiment. Bars represent average over these experiments and error bars the SEM. Statistical significance is denoted by * where *p* ≤ 0.05 determined by Mann–Whitney. **E** IHC representative images for CRM1 staining in OE and EV7 derived tumours, scale bar represents 100 μm. **F** Analysis of IHC images for CRM1. Graph displays average percentage of positively stained cells over at least three samples with error bars giving SEM. Statistical significance, where found, denoted by * with *p* ≤ 0.05 determined by Mann–Whitney. **G** Immunofluorescence of 3D spheroids, representative images for EV7 and OE19 clones grown in 3D, fixed and stained for the nuclear stain DAPI (Blue), CRM1/Alexa 555 (Red) and Cell volume with cell mask (green). **H** CRM1 nuclear to cytoplasmic ratio, determined through Volocity image analysis. Represents three independent experiments and five images from each experiment, bars represent average over these experiments and error bars the SEM. Statistical significance is denoted by * where *p* ≤ 0.05 determined by Mann–Whitney. **I** Immunoblot analysis of 3D spheroids grown in the presence or absence of cyclohexamide. Probed for CRM1 with loading control HSC70, image representative of three independent experiments. **J** Schematic outlining hypothesis for PPARG effect on: AKT3, PGC1α and mitochondrial biogenesis.

AKT3 has been shown to increase mitochondrial biogenesis through blocking PGC1α export from the nucleus via CRM1 [15]. It should be noted that the localisation of PGC1α within the cell rather than just the absolute abundance is important to its functionality [15]. Using immunofluorescence (IF) it is possible to observe PGC1α localisation within the cells of 3D spheroids and quantify this (Fig. 4A, B and Supplementary Fig. 3I), revealing that OE19 spheroids display a higher nuclear to cytoplasmic ratio of PGC1α than the EV7 control (Fig. 4B). To determine if this effect was truly dependent on AKT3, the experiment was repeated with knock down of *AKT3* with siRNA (Fig. 4C, D and Supplementary Fig. 3J). Knockdown of *AKT3* resulted in a loss of total PGC1α in both EV7 and OE19 (Fig. 4D). For EV7 this total loss did not affect the nuclear to cytoplasmic ratio, however for OE19 there appeared to be a shift from nuclear localisation to cytoplasmic (Supplementary Fig. 3J). This is consistent with increased CRM1 function resulting from loss of AKT3 suppression and consequently greater PGC1a export from the nucleus.

However, when examining the levels of CRM1 in tumour samples and spheroid lysates we observed that CRM1 levels appeared similar or higher in OE19 samples as compared to EV7 control (Figs. 2K and 3C). IHC analysis was performed for CRM1 on the tumours, which showed reduced nuclear CRM1 levels in the all OE clones as compared to EV7 derived tumours (Fig. 4E, F). AKT3’s regulation of CRM1 is through its ability to inhibit its function through phosphorylation of the CRM1 protein, inducing its degradation [15]. Such regulation may not affect the total level of protein but still affect functionality. To investigate this IF analysis of 3D spheroid cultures was performed (Fig. 4G), with the localisation of the CRM1 found to be altered in OE19 as compared to EV7. In the OE19 clones, cytoplasmic aggregates of CRM1 were observed, which were absent in the EV7 spheres, altering the nuclear to cytoplasmic ratio for CRM1 significantly (Fig. 4H). Although total CRM1 levels may not change, or may indeed increase, its location is altered, reducing its nuclear export function.

Furthermore, upon cycloheximide block of translation for twenty four hours, the known half-life of CRM1 [27], a dramatic reduction in CRM1 levels in the OE clones as compared to EV7 is observed (Fig. 4I and Supplementary Fig. 3K). This suggests that degradation of CRM1 is elevated in the OE clones, but the rate of protein production is high enough that the total level of protein appears unaffected or even elevated, whilst its localisation and thereby function is significantly altered.

PPARG through AKT3 is able to increase the nuclear levels of PGC1a, by disrupting the stability of nuclear export protein CRM1. AKT3 reduces the stability of CRM1 and its nuclear localisation, preventing its function and ensuring PGC1α remains in the nucleus. With increased nuclear PGC1α mitochondrial biogenesis and ETC complex levels are increased, which allows for the increase in ATP observed (Fig. 2G), fuelling growth and metastasis. When PPARG levels fall, the reverse is true, resulting in mitochondrial dysfunction [12–15, 25] and reduced ATP levels (Fig. 4J).

## Discussion

We have previously identified PPARG as a potential target in metastatic PC [4]. In this work we have been able to identify a novel effector of PPARG, AKT3, which elicits its effects through the AKT3-PGC1α axis. PPARG, traditionally thought as a regulator of lipid metabolism, was observed to up-regulate AKT3 which de-stabilises CRM1 increasing PGC1α retention in the nucleus. This leads to increased mitochondrial biogenesis and ETC levels. Consequently, ATP levels are elevated likely providing for the increased energetic demands of tumour growth and metastasis.

It appears that the role of PPARG within the prostate tumour environment is critical for survival within the in vivo system due to the inherent pressures and stresses of the tumour environment. Tumours lacking any PPARG expression fail to develop, and any tumours that do develop express PPARG (Fig. 1 and Supplementary Fig. 1). This would suggest that the tumour environment exerts a selective pressure on the tumour cells favouring those that express PPARG. The KO1 and KO2 cell lines in vitro express no PPARG (Fig. 1A), yet in vivo expressed equivalent levels to the NTS control (Fig. 1E and Supplementary Fig. 1A). A similar effect was observed in the OE clones, where EV7 developed a few small tumours, yet once again all of these were PPARG positive. During the same time period clone OE19 was able to develop end-point tumours in all but one mouse, clearly indicating a requirement for PPARG for in vivo prostate tumour development. Seahorse stress test assays identified a reduced mitochondrial function in the PC3-M KO cells when PPARG levels were reduced, yet, DU145 OE clones showed no differences in OCR. This was also true for a number of 2D in vitro assays performed (data not shown) for both DU145 OE and PC3-M KO cells, suggesting optimal growth conditions in 2D culture are not sufficiently representative of the stresses of the in vivo environment for the advantages of PPARG expression to be evidenced. Seahorse assays provide a slightly elevated level of stress due to the reduced serum concentration required, which may account for the observed effect in the PC3-M KO cells, yet this level of stress was not sufficient to demonstrate the effects of PPARG over-expression in DU145 cells. PPARG only appears to become functionally essential in vivo and in 3D culture conditions, which are more physiologically relevant.

The FASN (Fig. 1E, J, K and Supplementary Fig. 1C, D, J) and Seahorse data suggested a metabolic effect of PPARG, which influenced mitochondrial mass (Fig. 2E, F, K) and ATP levels (Fig. 2G). Given the known function of PPARG we anticipated this would result from up-regulation of genes involved in lipid metabolism. Surprisingly data from RNA-Seq of the tumour samples identified AKT3 (Fig. 2H) as a novel target of PPARG and the probable link between PPARG and the observed changes at the mitochondrial level given the known role of AKT3 in PGC1a regulation and mitochondrial biogenesis [14, 15, 25]. Indeed, using the eukaryotic promotor database two PPARG binding motifs are predicted within the AKT3 gene promotor, suggesting a possible mode of action for PPARG on AKT3 [28]. There are many regulators of AKT3; including VEGF, PDGF and IGF1, which may also play a role in AKT3’s function in PC, perhaps either in addition to or in synergy with PPARG and provide interesting avenues for future investigation [14, 29–31].

Growing the OE clones as spheroids highlighted a very striking phenotypic differences between the OE clones and their EV7 control, suggestive of a loss of cellular polarity, reminiscent of an EMT like phenotype (Fig. 3A, B and Supplementary Fig. 2G). Knockdown of AKT3 reduced this effect significantly, but not completely (Fig. 3D–F), suggesting perhaps in addition to its role in the AKT3/PGC1α pathway PPARG may also be affecting other pathways. This is supported by the data from the PPARG inhibitor and siRNA studies showing a more pronounced reversion of phenotype (Fig. 3G–I and Supplementary Fig. 2H–K). As the inhibitor and siRNA are acting on PPARG and therefore upstream of AKT3 it not only reduces PPARG’s effect on AKT3 but also on any other potential downstream effectors, resulting in the more pronounced effect

PPARG is a ligand activated nuclear receptor known for its roles in adipocyte differentiation and lipid metabolism. Its activity is governed by ligand binding, cellular localisation and post translational modifications the latter appearing to have an inhibitory role [32–34]. In our hands its activity was evidenced through increases in downstream effectors of lipid metabolism (Fig. 1E, J, K and Supplementary Fig. 1C, D, J) [4]. These lipogenic enzymes are observed by others to be regulated in a PGC1α dependant pro-tumourgenic manner [35]. Therefore, it is possible that these additional functional effects of PPARG and PGC1a are, along with PPARG’s ability to increase exogenous fatty acid uptake [36, 37], synergistic with the mitochondrial biogenesis effects of PPARG, and could account for some additional functions of PPARG in prostate cancer not attributable to AKT3.

The effect of PPARG driven AKT3 up-regulation results in an accumulation of PGC1α (Fig. 2K), which drives mitochondrial biogenesis and tumour growth and development [7, 8]. This appears to be at odds with the work from previously published studies [38, 39], which describe how PGC1α can supress PC metastasis and aggressiveness. In the Torrano et al. study PGC1α up-regulation drives oestrogen-related receptor alpha (ERRα) target gene transcription instigating a metabolic switch to a catabolic program, which inhibits PC progression [38]. This effect is almost completely dependent upon the transcription factor ERRα, a PGC1α co-factor. Similarly, the Kaminski et al. study links PGC1α’s suppressive effects to ERRα and c-Myc and downstream polyamine synthesis [39]. However, PGC1α is a co-activator for numerous transcription factors, such as PPARG and the other PPAR’s (alpha and beta), as well as ERR alpha, beta and gamma, Fox01, hepatocyte nuclear factor 4α (HNF4α) and nuclear respiratory factor 1 (NRF1) [6, 8, 40–44]. Notably both these studies [38, 39] focus on the PGC1α/ERRa relationship and the subset of genes they regulate as the cause of PGC1α suppression of metastasis. The PPARs and ERRs (with PGC1α) are known to regulate different gene sets [40] and perhaps it is this difference in the genes transcribed that determines if PGC1a is pro or anti-tumour progression. Where PPARG levels are high, as in our case, perhaps it is the effect of PGC1α rather than ERRα which is determining the gene signature.

Indeed, examining PGC1α levels in vivo by immunoblot revealed it to be elevated in OE tumours. IF analysis of 3D spheroids showed the nuclear localisation of PGC1a to be similarly affected (Fig. 4A, B). This effect appeared to be lost upon knockdown of AKT3, which may explain the phenotypic effects observed on spheroid growth upon AKT3 knockdown observed earlier (Fig. 4C, D). CRM1 however, initially appeared to contradict the hypothesis. For PGC1α to remain nuclear a reduction in CRM1 levels was anticipated in conjunction with the increased AKT3 function driven by PPARG.

The action of AKT3 upon the major nuclear export protein CRM1, affects the subcellular localisation of PGC1α. Given AKT3’s classification as a Serine/Threonine Kinase, it is suggested its effects on CRM1 could be via phosphorylation [15, 45]. Indeed serine 1054 of CRM1 was identified as crucial to AKT3’s ability to inhibit CRM1 [15], however no evidence of AKT3 directly phosphorylating this site has yet been observed. IHC analysis for CRM1 showed significantly reduced CRM1 levels in OE18 and OE19 (Fig. 4E, F), but immunoblotting did not appear to reflect this (Figs. 2K and 3C). Performing IF for CRM1 on the 3D spheroids (Fig. 4G, H) demonstrated large non-nuclear aggregates of CRM1 were present in clone OE19 and not in EV7. For CRM1 to export PGC1a from the nucleus it must be localised to the nucleus. The presence of these aggregates’ accounts for the lack of change in CRM1 levels whilst allowing for PGC1α to remain in the nucleus at a higher level than in EV7 cells. Blocking translation using cycloheximide, confirmed that indeed in the OE clones CRM1 was being degraded at an elevated rate (Fig. 4I), suggesting that PPARG through AKT3 affects the stability, and consequently function of the CRM1 protein and thereby retention of PGC1α in the nucleus.

Here we provide evidence for a previously unidentified functional link between PPARG-AKT3-PGC1a, mitochondrial biogenesis and mitochondrial output (Fig. 4J). This function of PPARG allows for the provision of energy (ATP) which may fuel tumour growth and progression in PC. The failure of PPARG negative tumours to develop suggests that PPARG is essential for tumour growth and progression in vivo. This absolute requirement for PPARG highlights its crucial role in PC development, suggesting that targeted therapies against PPARG could prove to be a highly efficient avenue for treatment of advanced PC.

## Supplementary information

Supplementary Figure Legends

Supplementary Methods

Supplementary Figure 1

Supplementary Figure 2

Supplementary Figure 3
