# Supplementary material for: PPAR-gamma induced AKT3 expression increases levels of mitochondrial biogenesis driving prostate cancer
Source: Oncogene. 2021 Mar 2;40(13):2355–66. doi: 10.1038/s41388-021-01707-7 (PMC8016665; doi:10.1038/s41388-021-01707-7)
Supplement: Supplementary file 1 — Supplementary Figure Legends [file 41388_2021_1707_MOESM1_ESM.docx]

**Supplementary Figure 1: (A)** Analysis of IHC images for PPARG, from KO and Scrm control derived tumours. Graph displays average percentage of positively stained cells over at least three samples with error bars giving SEM. Statistical significance, where found, denoted by * with p≤0.05 determined by Mann Whitney. **(B)** IHC representative images for Ki67 staining in KO and Scrm control derived tumours, scale bar represents 100μm. **(C)** Analysis of IHC images for Ki67, PPARG & FASN from KO and Scrm control derived tumours. Graph displays average percentage of positively stained cells over at least three samples with error bars giving SEM. Statistical significance, where found, denoted by * with p≤0.05 determined by Mann Whitney. **(D)** Spearman correlation of PPARG and FASN expression levels by IHC in KO and Scrm derived tumours. Spearman r=0.7111, p<0.001 by non-parametric (spearman) rank correlation. **(E)** IHC for RFP in all KO tumours. Positive control for RFP is mouse kidney expressing RFP. **(F)** RNA-scope *in situ* hybridisation representative images for human PPIB probe, scale bar represents 100μm. **(G)** Halo software analysis of RNA-scope for Human PPIB on tumours, and corresponding non tumour tissue derived from prostates of mice implanted with OE & EV7 cell lines. Analysis performed on all remaining material for each cell type used, graph displays average with error bars giving SEM. Statistical significance denoted with * where p≤0.05 determined by Anova and Dunnett's multiple comparisons test. **(H)** IHC representative images for Ki67 staining in OE and EV7 control derived tumours, scale bar represents 100μm. **(I)** IHC image analysis for Ki67 for OE and EV7 control derived tumours. Graph displays average percentage positive cells over at least three samples per clone with error bars giving SEM. Statistical significance, where found, denoted by * with p≤0.05 determined by Mann Whitney. **(J)** Spearman correlation of PPARG and FASN expression levels by IHC in EV7 and OE derived tumours. Spearman r=0.6427 with p<0.001 by non-parametric (spearman) rank correlation **(K)** Halo analysis of IHC images for PPARG, from Scrm control & OE19 derived tumours. Graph displays average percentage of positively stained cells over at least three samples with error bars giving SEM. Statistical significance, where found, denoted by * with p≤0.05 determined by Mann Whitney.

**Supplementary Figure 2:** **(A)** Full list of statistically significant hits form RNA-scope on tumour samples, analysis as described in material and methods. Statistical significance increases the further up the heat map, with statistical significance threshold of p≤0.05. **(B)** Immunoblot analysis from tumours derived from EV7, OE12 and OE18 orthotopic injected cells. Showing results for PPARG and loading control HSC70. Values below each band indicate the densitometry value of the band as normalised to the loading control HSC70 and compared to the first EV7 band. **(C)** Immunoblot analysis from tumours derived from EV7, OE12 and OE18 orthotopically injected cells. Showing results for PGC1α, the complexes of the ETC, AKT3, VDAC1 and loading control HSC70. For the ETC complexes, complex I is labelled in blue and complex IV in red to allow the reader to identify the correct densitometry value for the corresponding bands, image representative of three independent experiments. Values above each band indicate the densitometry value of the band as normalised to the loading control HSC70 and compared to EV7.

**Supplementary Figure 3:** **(A)** Immunoblot analysis of lysates from 3D spheroid growth of EV7, OE12, OE18 & OE19 for EMT markers N-Cadherin & Vimentin, with HSC70 as loading control. Values below each band indicate the densitometry value of the band as normalised to the loading control HSC70 and compared to EV7. Image representative of three independent experiments. **(B)** Representative images of the characterisation of the phenotypic effect of PPARG over-expression in in OE12 and OE18 compared to EV7 cells. **(C)** Quantification of the area of the spheroids in OE12 and OE18 compared to EV7, three independent experiments with ten technical replicates per experiment, Statistical significance denoted by * p≤0.05 determined by Mann-Whitney. **(D)** Immunoblot analysis of lysates from 3D spheroid growth of EV7 and OE12& 18, for AKT3, VDAC, and the complexes of the ETC, HSC70 used as loading control. For the ETC complexes, complex I is labelled in blue and complex IV in red to allow the reader to identify the correct densitometry value for the corresponding bands, image representative of three independent experiments. Values above each band indicate the densitometry value of the band as normalised to the loading control HSC70 and compared to EV7. **(E)** siRNA knockdown of PPARG effect in 3D culture appearance on EV7 and OE19 cells grown in 3D either treated with non-targeting siRNA (siNTS) or PPARG targeting siRNA (siPPARG). Images representative of three independent experiments **(F)** Quantification of the two different spheroid appearances following PPARG knockdown, represents three independent experiments, ten technical replicates, Statistical significance denoted by * p≤0.05 determined by 2-way Anova and Sidak’s multiple comparisons test. **(G)** qPCR analysis for PPARG from 3D lysates following siRNA treatment, either siNTS or siPPARG, three independent experiments with three technical replicates per experiment, error bars show SEM. **(H)** Immunoblot analysis from lysates derived from 3D siRNA knockdown of PPARG, showing the ETC complexes, VDAC1 and AKT3. HSC70 was used for loading control. Image representative of three independent experiments. **(I)** Quantification of total PGC1a levels by IF in 3D culture in EV7 and OE19 clones normalised to cell number. Four independent experiments and six images from each experiment. Bars represent average over these experiments and error bars the SEM. **(J)** PGC1α nuclear to cytoplasmic ratio after AKT3 knockdown, determined through Volocity image analysis. Represents three independent experiments and five images from each experiment, bars represent average over these experiments and error bars the SEM. **(K)** Immunoblots for CRM1 following cyclohexamide treatment for the times indicated above bands. Values below bands indicate the densitometry value of the band as normalised to the loading control HSC70 and compared to zero hour band for each clone. Images representative of three independent experiments.
