# Supplementary material for: PPAR-gamma induced AKT3 expression increases levels of mitochondrial biogenesis driving prostate cancer
Source: Oncogene. 2021 Mar 2;40(13):2355–66. doi: 10.1038/s41388-021-01707-7 (PMC8016665; doi:10.1038/s41388-021-01707-7)
Supplement: Supplementary file 2 — Supplementary Methods [file 41388_2021_1707_MOESM2_ESM.docx]

**Supplementary Methods**

**Cell Culture**: Human prostate cancer cell lines PC3-M and DU-145 were grown in RPMI (Gibco), containing 10% serum supplement and 2mM L-Glutamine at a temperature of 37^0^C with 5% CO2. These cells have been tested for and found to be negative for mycoplasma contamination and authenticated by LGC standards.

PC3-M cells were transfected with CRISPR/CAS9 constructs for *PPARG*: PPARG HDR plasmid and PPARG CRISPR/CAS9 KO plasmid (sc-422363-HDR, sc-422363 Santa Cruz Biotechnology, Inc) using nucleofection (Lonza kit V). Puromycin selection was applied and clones picked following selective pressure. Three clones were identified to have the desired loss of PPARG at the mRNA and protein levels and these were used throughout the paper. Control clones were generated by nucleofection using the Control CRISPR/CAS9 plasmid, (sc-418922 Santa Cruz Biotechnology, Inc) and piRFP-IRES-puro plasmid (to control for the puromycin selection effect) [1]. One clone was selected following puromycin selection to act as the control. All clones were maintained under puromycin selection when in culture.

DU-145 cells were transfected with either PPARG (Myc-DDK-tagged)-Human peroxisome proliferator-activated receptor gamma (PPARG), transcript variant 1 in pCMV-Entry vector (origene, RC201538) or the empty vector pCMV6-Entry (origene, PSI00001). The former following G418 antibiotic selection generated clones over-expressing PPARG and the latter was used to generate control clones following G418 selection. Again, three clones were selected following selective pressure and one control clone. All were maintained under G418 selection in culture.

**Orthograft surgical procedure, monitoring and sample harvesting:** Mice were anaesthetized by isoflurane inhalation (with vaporiser set for 5% for induction and the 3% for maintenance) and then a midline lower abdominal incision was made. Cells were then injected into the one of the anterior prostate lobes using a 1-cc syringe with a 27-gauge needle. For PC3-M background clones 1x10^7^ cells were injected into each mouse. For DU-145 background clones 2x10^6^ cells were injected into each mouse. DU-145 cells were injected in a suspension of serum and phenol red free, glutamine containing RPMI, no antibiotic, 50μl per injection. PC3-M cells were injected in the same media formulation but with the addition of 25μl Matrigel, and therefore only 25μl media. Successful injection was judged on the formation of a bleb within the prostate lobe. The abdominal cavity was closed with surgical sutures and the skin closed with surgical staples. Six mice were used per clone injected, however due to technical difficulties only five mice were used for the KO1 and KO2 cohorts two had to be censored.

Mice were given Vetergesic (dose defined by assigned veterinarian) following anesthesia prior to beginning surgery, and a second dose administered the following day. In addition, local anesthetic Marcaine (dose defined by assigned veterinarian) was given during surgery to abdominal area.

Two weeks following surgery mice began to be monitored using ultrasound (Vevo 3100 from Fujifilm VisualSonics). Mice were anaesthetized by isoflurane inhalation then subjected to ultrasound on the lower abdomen to identify and monitor emerging tumours. After six weeks some mice exhibited tumours that had reached a size (not greater than 1.5cm diameter) such that the experiment had to be terminated, as such all mice were euthanized at this point.

Blood and other organs of interest were harvested including lymph nodes and liver and either snap frozen and stored in a -80°C freezer for further processing or fixed in formalin for 48 hours prior for histological processing. Harvested prostates were weighed then divided in half; one half sent for histological processing following 48 hours formalin fixation. The other half was snap frozen on dry ice and later transferred to the -80°C freezer, prior to further processing.

**Extraction of RNA from tissue samples:** RNA was extracted from snap frozen tissue by firstly pulverising the tissue using a micro-homogenizer (three tumour tissue samples per clone with the exception of KO2 where only two samples were used as this was all that was available). The powdered tissue was then re-suspended in RLT buffer (Qiagen RNeasy Mini Kit) and further homogenized using Precellys tubes and Precellys Evolution homogenizer (Bertin Instruments). Once homogenized the RNA was extracted using the Qiagen RNeasy Mini Kit, as per the manufacturer’s instructions, including the DNase digestion step.

**Real Time PCR (RT-PCR):** RNeasy mini kit (Qiagen) was used to isolate RNA from both cell lines, using the manufacturer’s protocol. 5μg RNA was used for cDNA synthesis using High Capacity cDNA reverse transcription kit (Applied Biosystems). PCR was performed in the 7500 Fast Real-Time PCR system (Applied Biosystem), using TaqMan^®^ Gene expression Master Mix (life technologies). Primers were designed using Universal Probe Library Assay Design Center (Roche) and the respective probes used from the Universal probe library. Actin was used as reference gene for normalisation and the relative quantities determined by normalizing to a biological control sample as indicated in each figure. Results are from three biological replicates with three technical replicates each.

Primers and probes combinations

PPARG: Fwd (5′-gacaggaaagacaacagacaaatc-3′), Rev (5′-ggggtgatgtgtttgaacttg-3′) #7

*ACTIN*: Fwd (5’- ccaaccgcgagaagatga-3’) Rev (5’-ccagagcgtacagggatag-3’) #64

*CACS3*: Fwd (5’-ggggttccagttaatacaagtttc-3’ Rev (5’-gccagctgtatttctcttctgag-3’)_#84

# Immunoblotting: Cells and pulverised tissue were lysed in lysis buffer (50 mM Tris pH 7.6, 150 mM NaCl, 1% Triton X-100, 0.5% deoxycholate, 0.1% SDS, 1 mM sodium ortho-vanadate, 5 mM sodium fluoride and protease inhibitor cocktail, Calbiochem) for cells lysis was performed at room temperature. For tissue lysis brief sonication was also used to aid with complete lysis at 4°C.

# For 3D samples spheroids and matrigel were manually homogenized in the culture media by scratching with a pipette tip and transferred into an Eppendorf where they were centrifuged at 300xg to separate the cells from the Matrigel. Media and Matrigel were then aspirated off and the remaining pellet washed twice with PBS and the above process repeated before lysis buffer was applied.

# Protein concentrations of lysates were quantified using the Pierce^TM^ BCA protein assay kit (as per manufactures instructions) and then resolved by SDS/PAGE on 4-12% gradient polyacrylamide gels (Invitrogen) at 150 V for 2 h and transferred electrophoretically onto PVDF membranes (Milipore) at 28V for 2h. Blots were blocked for 30 min with 5% skimmed milk in TBST and probed with the respective antibodies (in 5% skimmed milk, NaA_2_ in TBST) overnight at 4^0^C. After incubation with HRP conjugated secondary antibody, bands were detected using ECL (GE) detection reagent or Pierce™ ECL Plus Western Blotting Substrate.

# Immunoblotting was performed with the following antibodies: PPARG (Cell Signalling #2435), ACTIN (Sigma, A4700), AKT3 (Cell Signalling #14982), HSC70 (Santa Cruz Biotechnology, SC-7298), Total OXPHOS human WB antibody cocktail (abcam, ab110411), VDAC1 (abcam, ab15895), PGC1a (abcam, ab54481), CRM1 (Cell signalling #46249), N-Cadherin (Cell signalling #13116), Vimentin (Cell signalling #5741), Anti-rabbit IgG, HRP linked antibody (Cell signalling, # 7074) and Anti-mouse IgG, HRP linked antibody (cell signalling #7076). For all immunoblots images shown are representative of three independent biological replicates.

**Immunohistochemistry & RNA-scope:**

Immunohistochemistry

Formalin fixation and paraffin embedding

Immediately following dissection tissue was fixed in 10% neutral buffered formalin at room temperature overnight. After fixation, tissues were dehydrated and embedded in paraffin blocks for subsequent sectioning and staining. H&E staining was performed routinely on tissues to assess general histopathology.

The following antibodies were investigated with immunohistochemistry (IHC); FASN (Cell Signalling, 3180), Ki67 (Thermo, RM-9106), PPARG (Abcam, ab59256), VDAC1 (Cell Signalling, 4866**)** CRM1 (Cell Signalling, 46249).

Approximately 4 μm sections of formalin–fixed paraffin–embedded tissues were cut, mounted on adhesive slides and placed in a 60°C incubator overnight. Sections were dewaxed for 5 minutes in xylene before rehydrating through decreasing concentrations of alcohol followed by washing with H_2_O for 5 minutes. Tissue sections underwent heat–induced epitope retrieval in a Dako pre-treatment module. Briefly, sections were heated in 10 mM sodium citrate retrieval buffer, pH 6 (Thermo Scientific, catalogue no. TA250PM1X) for 25 minutes at 98°C before cooling to 65°C. Slides were then removed and washed in Tris Buffered Saline with Tween (Thermo Scientific, catalogue no. TA999TT) before undergoing antibody staining on the Dako Autostainer Link 48 platform at previously mentioned optimised dilution using Dako Rabbit Envision (K4003) to bind to the antibody and 3,3’ diaminobenzidine (Dako, K3468) to visualise the antibody-antigen complex. Finally, slides were counterstained with haematoxylin and dehydrated in increasing concentrations of alcohol, then taken through 3 changes of xylene prior to sealing with glass coverslips using DPX mountant for microscopy.

The antibodies below were stained using the protocol defined above for the Dako 48 autostainer link at the dilution mentioned -

FASN 1/30

Ki67 1/100

PPARG 1/75

Leica Bond Rx autostainer: The appropriate sections were loaded onto the Leica Bond Rx autostainer. The sections were retrieved using Leica ER2 (AR9640) retrieval buffer for 20 minutes at 95°C. The antibodies were used at the mentioned previously optimised dilution using Dako Rabbit EnVision to bind to the antibody and 3,3’ diaminobenzidine to visualise the antibody-antigen complex. Sections were counterstained with haematoxylin on board the autostainer, dehydrated in increasing concentrations of alcohol, then taken through 3 changes of xylene prior to sealing with glass coverslips using DPX mountant for microscopy.

The antibodies below were stained using the protocol detailed above for the Leica Bond Rx autostainer at the dilution mentioned -

CRM1 1/250

VDAC1 1/40

RNAscope:

*In situ-hybridisation* detection for *Hs-PPIB* (313908; Advanced Cell Diagnostics, Hayward, CA;) and *AKT3* (Advanced Cell Diagnostics, Hayward, CA; 434218) mRNA was performed using RNAscope 2.5 LS (Brown) detection kit (Advanced Cell Diagnostics, Hayward, CA; 322100) on a Leica Bond Rx autostainer strictly according to the manufacturer's instructions. Staining was performed on 4um formalin fixed paraffin sections which had been cut and placed in a 60°C oven for 2 hours prior to staining.

IHC quantification

All IHC slides were scanned using a Leica SCN 400 F scanner at X20 magnification. Areas of tumour epithelium were scored according to the percentage of positive cells in each high-power field. Six mice were used from each clone (with the exception of K01 and K02, where only five mice were used), with 3 high-powered fields scored from each slide.

RNA-scope quantification by HALO

All RNA-Scope slides were scanned using a Leica SCN 400 F scanner at X40 magnification, images were uploaded to Halo Image analysis platform (Indica labs.). RNA-scope derived images were analysed using the ISH v2.2 algorithm, and analysis performed only upon the annotated layer which was drawn around the tumour mass. Results were given as number of probe copies per tissue area. Six mice were used from each clone, with the whole tumour area on each slide being scored.

**Seahorse Bioscience Assay**

All clones were assayed for mitochondrial function using the Seahorse Flux Analyser. Method as previously described[2]. Briefly, cells were seeded at 40,000 cells/well for DU-145 cells and 50,000 cells/well for PC3-M cells in a XF96 cell culture micro plate (Seahorse bioscience) in 80µl of DMEM, supplemented with 10%FCS and 1% L-glutamine and left to attach overnight at 37°C under 5% CO_2_. Four wells were left with no cells to serve as background wells for the assay.

On the day of assay 200µl of XF calibrant was applied to every well of the cartridge plate of an XF96 Assay Kit (Seahorse Bioscience) and placed at 37°C in a non-gassed incubator for a minimum of 1 hour. During this time the Seahorse assay media was warmed to 37°C and supplemented with; 10% glucose, 1% FBS, and pH adjusted to 7.4. Oligomycin, CCCP, antimycin and rotenone were diluted in the seahorse media to concentrations of 1µM, 0.25 µM and 0.5 µM respectively for both cell types. Each drug was then applied to the appropriate ports in the sensor plate 25µl per port. The DMEM on the cells in the cell culture plate was removed and replaced with 150µl of pre-prepared seahorse media. The cell plate was then transferred to the non-gassed 37°C incubator to equilibrate for at least 30 minutes. The Seahorse protocol begins with calibration of the sensor plate; on completion the cell plate will have finished equilibrating and is loaded to complete the protocol. The measurement protocol was the same for all assays; 4x mix/wait/measure steps to give four basal readings for oxygen consumption rate (OCR) and extracellular acidification rate (ECAR). The first port was then injected and the 3x mix/wait/measure protocol repeated to get OCR and ECAR readings based on the effect of the drug. This was repeated for all ports and measurements for all drugs in succession.

Following assay completion, the cells were fixed within cell culture plate by adding 28µl 10% Trichloroacetic acid (TCA)/well. The plate was then incubated at 4°C for 1 hour, following which the TCA/media solution was removed and the plate washed with water. The plate was then air-dried, then 28µl/well of 0.04% sulforhodamine B (SRB) applied to each well and incubated for 30 minutes at room temperature. Excess is then removed, and plate washed until running clear in 1% acetic acid. Then left to air-dry overnight. The SRB stain is then eluted from the cells using 100μl/well 10mM TRIS pH10.5. 50μl is added to a fresh well of a 96 well plate along with a further 200μl 10mM Tris pH10.5 for quantification. SRB staining is a well-established colorimetric protein assay and based on standard curve of cell number versus SRB absorbance at 510nm the data from the seahorse can be normalised on a well by well basis to cell number, using Molecular Devices SpectraMax 384 spectrophotometer [3]. Three independent experiments were performed, with four technical replicates for each clone.

**Carbon 13 3D metabolomics:**

Cell preparation Cells were split 1:10 in 2D culture the day prior to seeding in 3D. These cells were then trypsinized and counted, and aliquoted in volumes to give 60,000 cells. These were then centrifuged to pellet the cells and trypsin removed and pellet resuspended in 100% growth factor reduced Matrigel (Corning, 354230) being careful to not make bubbles. Using a 24 well plate 20μl of the cell Matrigel mix is seeded into the centre of the well, this is incubated at 37°C briefly before 500μl of standard cell culture media is added and cells incubated overnight. The following day the medium was removed and replaced with fully labelled ^13^C glucose containing medium (Cambridge Isotope Laboratories, #110187-42-3) and cells were then harvested 8 hours later. Details on metabolite extraction and LC-MS analysis are described previously [4]. Three biological replicates were performed for each clone, with three technical replicates per biological replicate.

To normalize the data, after metabolic extration Matrigel/cell plugs were lysed with lysis buffer for a few minutes then the Matrigel plug scratched apart with a pipette tip to allow for full lysis. The contents of the well were then transferred to a Precylls tube and homogenized by Precylls Evolution homogenizer (Bertin Instruments). Following homogenisation the sample was left to let bubbles settle and then 10μl of resultant lysates were used to perform a BCA assay (Thermo Scientific/ Pierce, as per manufactures instructions) to give a protein concentration for each well to enable normalization of the metabolic data to protein.

**3D cell culture:**

Cells were split 1:10 in 2D the day before setting up 3D culture. On day of set up of the 3D culture the previously set up 2D culture was trypsinized and centrifuged to remove trypsin and re-suspended in 5ml of culture medium. Cells were counted and adjusted to give 1.25X10^5^cells/ml. A 6 well plate was coated in 180μl/well growth factor reduced (GFR) Matrigel (Corning), then incubated at 37°C for 15 minutes. To coated wells 1.6ml of cell suspension was added with 2% GFR Matrigel. When cultured for immunoblotting, cells were, as above, split 1:10 in 2D the day before setting up the culture. However, on day of set up of the 3D culture the previously set up 2D culture was trypsinised to detach from the culture vessel and counted. Then the desired volume (to give 60,000 cells/well) of cells was centrifuged to remove trypsin. The cell pellet was then re-suspended in Matrigel (to give 20μl Matrigel/per well). Then 20μl of Matrigel/cell suspension (containing 60,000 cells) was plated as a drop into the middle of a well of a 24 well plate. This was placed in a cell culture incubator briefly at 37°C before culture 1ml culture medium was added to each well to cover the Matrigel drop. For every condition three wells were set up. Cultures were grown for three days before harvesting (as described above), at which point the three wells for each condition were pooled.

3D immunofluorescence

3D spheroids were cultured as described [5] and in supplementary information with the following differences. A glass bottomed eight well chamber slide was placed on an open 10cm dish on top of ice, and then each well coated in 60μl Matrigel and incubated at 37°C for 30 minutes. Meanwhile cells were counted and diluted to 2.5X10^4^cells/ml. 300μl of cell suspension with 2% Matrigel was added to the corresponding wells and incubated for 3 days before fixing.

For fixing wells were washed twice with PBS then fixed in 4% paraformaldehyde (PFA) for 15 minutes at room temperature. Fixed cells were then washed briefly with PBS to remove the PFA and then permeabilized with 100% ice cold methanol for 10 minutes at -20°C. Methanol was then removed and wells blocked using a 1%BSA, 0.3% triton solution in PBS for 1 hour. Primary antibody was then applied in blocking solution for overnight incubation at 4°C on a very slow rocker. This was removed the following day and cells washed three times in blocking solution at room temperature before secondary antibody was applied in blocking solution along with the cell mask green stain (ThermoFischer) at a 1:50000 dilution and incubated for 1 hour at room temperature. Cells were washed with PBS three times at room temperature before Vector Shield containing DAPI (vector laboratories) was added to stain the nuclei and seal the fixed samples. These were stored at 4°C protected from light until imaged.

Antibodies used and dilutions below:

PGC1a (Novus NBP1-04676): 1:1000

CRM1 (Cell signaling #46249): 1:400

Rabbit Alexa Fluor 555: 1:200

**Analysis of 3D imaging data:**

Analysis of 3D immunofluorescence:

Fixed samples were imaged on the NIKON A1R confocal microscope using a Plan Fluor 40x Oil DIC H N2 lens or a Plan Apo 20x Dry DIC M N2 lens. Both single slice and three-dimensional Z-stack images were taken using the 405nm, 488nm and 560nm laser to excite the blue (DAPI), Green (Cell mask) and Red (AlexaFluor 555) respectively. Four biological replicates for the PGC1α IF were performed with five images for each clone analyzed. For the CRM1 and PGC1α (after siAKT3 treatment) IF, three biological replicates were performed with ten images for each clone analyzed. Single slice images were used for presentation purposes; Z-stack images were analyzed using Volocity image analysis software (Perkin Elmer). Briefly z-Stack images were loaded into Volocity software. Using the DAPI stain the nuclei were identified in every image. Using the AlexaFluor 555 stain the PGC1a or CRM1 levels in both the nucleus and cytoplasm could be quantified using the DAPI to define nuclear localization. . From these two values the ratio of nuclear:cytoplasmic PGC1a or CRM1 was calculated and normalized to cell number based on the nuclear/DAPI stain.

Analysis of Sphere vs. Projections: For analysis of the EV7 vs. OE19 cells grown in 3D, images were taken 3 days following seeding onto 3D matrix (described above). Images were taken using a Zeiss Axiovert25 fitted with a QiImaging Retiga Exi FAST1397 camera using a Zeiss CP Achromat 10x lens. Three independent replicates were performed for each experiment and for each replicate ten images were taken for each condition, giving in total 30 images for each condition. Each image was examined, and the percentage of spheres and projections counted.

Analysis of Spheroid area: For analysis of the size of the spheroids in OE12 and OE18 cells vs. EV7 spheroids. As above images were taken 3 days following seeding onto 3D matrix. Images were taken as described above. Image analysis was performed using a specifically designed IMAGEJ macro, details given below. Briefly, the macro was able to identify all spheroids within the image, excluded those that touched the edges of the image (to prevent artifacts in the measurements). It then created a perimeter around each spheroid before allowing a user interactive step to join or separate spheroids it could not distinguish as joined or separate itself. Following this it then measured and generated a list of measurements of the area of each spheroid identified by the perimeter created. These areas were averaged for each cell type over three independent experiments and ten images per experiment to give the data shown.

IMAGEJ Macro

// Macro Written by David Strachan for Laura Galbraith

// The object of this macro is to take Phase contrast TIF images

// Outline the Spheres, allow the user to manually modify the mask

// then measure each sphere individually and output the results to the screen.

// Address any questions to D.Strachan@Beatson.gla.ac.uk

// Copyright (C) 2018 David Strachan - All Rights Reserved.

// This software is provided 'as-is', without any express or implied warranty. In no event will the authors be held liable for any damages arising from the use of this software.

// Spheres_beta1.1

// Last updated 15-08-2018

//

// Set pencil tool width to 2 by double clicking on Tool in Toolbar

//initialisation

init();

minSize = 0.005; // Min size that an object can be to be counted.

ms = d2s(minSize,4);

run("Set Measurements...", "area redirect=None decimal=9");

filename = File.nameWithoutExtension;

// Check to see that the images are the same scale as used to design this macro.

if (checkScale() == 1){

setGlobalScale();

dir = File.directory;

createDir(dir);

rename("image");

run("Duplicate...", "title=workingCopy");

processImagePart1();

processImagePart2();

do {

// put up a menu for the user to choose

choice = menu();

if (choice == 0){

selectImage("workingCopy");

close();

roiManager("Select", 0);

roiManager("Split");

roiManager("select",0);

roiManager("Delete");

getRidSmall(minSize);

doMeasure();

}

if (choice == 1){

manualSplitJoin();

processImagePart3();

}

} while (choice != 0);

} else exit("Error this macro was designed to use images that have a different magnification");

saveResults();

exit("Your results have been saved in "+dir+File.separator+filename+File.separator);

function saveResults(){

run("Flatten");

saveAs("Jpeg", dir+File.separator+filename+File.separator+filename+"_overlay.jpg");

close();

roiManager("Deselect");

roiManager("Save", dir+File.separator+filename+File.separator+filename+"_RoiSet.zip");

saveAs("results", dir+File.separator+filename+File.separator+filename+"_Results.xls");

}

function doMeasure(){

// Measure the individual objects

for (loop = 0; loop < roiManager("count");loop++){

roiManager("select",loop);

roiManager("rename","Obj - "+ loop +1);

}

print("[Results]","\\Clear");

roiManager("deselect");

roiManager("measure");

selectImage("image");

roiManager("show all with labels");

}

function getRidSmall(minSize){

for (loop = roiManager("count")-1; loop > 0 ; loop--){

roiManager("select",loop);

roiManager("Measure");

a = getResult("Area",nResults()-1);

//print(a);

if (a < minSize){

roiManager("Delete");

}

}

}

function menu(){

Dialog.create("Spheres menu system");

//items = newArray("Accept current segmentation and Measure", "Split - Joined Objects","Join - Split Objects");

items = newArray("Accept current segmentation and Measure", "Manually Split or Join Objects");

Dialog.addRadioButtonGroup("Please Choose", items, 2,1,1);

Dialog.show();

choice = Dialog.getRadioButton();

for (i = 0; i < 2; i++){

if (items[i] == choice) return(i);

}

}

function processImagePart1(){

run("Bandpass Filter...", "filter_large=10 filter_small=0 suppress=None tolerance=5 autoscale saturate");

run("Smooth");

run("Find Edges");

run("Convert to Mask");

run("Analyze Particles...", "size="+ms+"-Infinity show=Masks exclude include in_situ");

}

function processImagePart2(){

run("Duplicate...", "title=edges");

run("Bandpass Filter...", "filter_large=5 filter_small=0 suppress=None tolerance=5 autoscale saturate");

setAutoThreshold("Default dark");

setOption("BlackBackground", false);

run("Convert to Mask");

run("Create Selection");

roiManager("Add");

selectWindow("workingCopy");

roiManager("Select",roiManager("count")-1);

run("Fill", "slice");

roiManager("Select",roiManager("count")-1);

roiManager("delete");

run("Select None");

run("Options...", "iterations=3 count=1 do=Erode");

run("Watershed");

run("Options...", "iterations=3 count=1 do=Erode");

run("Analyze Particles...", "size="+ms+"-Infinity circularity=0.00-1.0 show=Masks exclude include in_situ");

selectImage("edges");

close();

showOnImage();

}

function processImagePart3(){

roiManager("select", roiManager("count")-1);

roiManager("delete");

run("Select None");

run("Fill Holes");

run("Analyze Particles...", "size="+ms+"-Infinity circularity=0.00-1.0 show=Masks exclude include in_situ");

showOnImage();

}

function showOnImage(){

run("Create Selection");

roiManager("Add");

selectWindow("image");

roiManager("select", roiManager("count")-1);

}

function manualSplitJoin(){

selectImage("workingCopy");

run("Select None");

setTool("Pencil Tool");

makeColor("White");

waitForUser("Split Joining Objects","Please Split Joining Objects - Then click OK to continue");

makeColor("Black");

waitForUser("Join Split Objects","Please Join Split Objects - Then click OK to continue");

}

function makeColor(color){

if (color == toLowerCase("white")){

setForegroundColor(255,255,255);

}

else if (color == toLowerCase("black")){

setForegroundColor(0,0,0);

}

else if (color == toLowerCase("yellow")){

setForegroundColor(255, 255, 0);

}

else if (color == toLowerCase("red")){

setForegroundColor(255,0,0);

}

else if (color == toLowerCase("grey")){

setForegroundColor(127,127,127);

}

}

function createDir(dir){

if (!File.exists(dir+File.separator+filename)){

File.makeDirectory(dir+File.separator+filename);

}

}

function init(){

close("results");

close("Log");

while (nImages > 1){

selectImage(1);

close();

}

roiManager("reset");

roiManager("Show None");

makeColor("Black");

}

function getNumbers(){

newImage("measure", "8-bit white", getWidth(), getHeight, 1);

run("Set Scale...", "global");

is("global scale")

}

function checkScale(){

// If scale is set to 300 pixels per inch then return 1

getPixelSize(unit, pixelWidth, pixelHeight);

if (unit == "inches" && ((pixelWidth - 0.003333) < 0.000001)){

return (1);

}

else {

return (0);

}

}

function setGlobalScale(){

run("Set Scale...", "distance=300 known=1 pixel=1 unit=inch global");

}

**siRNA knockdown and inhibitor treatments of cells**

For knockdown cells were set up for 3D culture as described and, in accordance with the manufacturer’s instructions lipofectamine RNAiMAX was used in a ‘reverse reaction’ to transfect ON-TARGET plus SMARTpool siRNA specific to *AKT3* (Dharmacon L-003002-00-005), ON-TARGET plus SMARTpool siRNA specific to *PPARG* (Dharmacon L-003436-00-0005) or the ON-TARGET plus Non-targeting pool (Dharmacon D-001810-10-20) as a negative control, then cultured for three days before harvesting for immunoblotting or fixing for immunofluorescence (as described previously).

For PPARG inhibitor treatments, cells were set up for 3D culture as described previously and additionally given GW96622 at a final concentration of 20μM, or corresponding volume of 100% DMSO (the inhibitor vehicle) as a control. Then cultured for three days before imaging or harvesting for immunoblotting (as described above).

For cyclohexamide treatment, again cells were set up in 3D cultures as described above and additionally given cyclohexamide at a final concentration of 10μg/ml or a corresponding volume of sterile H_2_O (cyclohexamide vehicle), as a control. Then cultured for up to 24 hours before harvesting at the designated time points for immunoblotting.

References

1. Hock, A.K., et al., *iRFP is a sensitive marker for cell number and tumor growth in high-throughput systems.* Cell Cycle, 2014. **13**(2): p. 220-6.

2. Galbraith, L.C.A., *The Role of Cardiolipin in Mitophagy*. 2014, The University of Glasgow.

3. Vichai, V. and K. Kirtikara, *Sulforhodamine B colorimetric assay for cytotoxicity screening.* Nat Protoc, 2006. **1**(3): p. 1112-6.

4. Mackay, G.M., et al., *Analysis of Cell Metabolism Using LC-MS and Isotope Tracers.* Methods Enzymol, 2015. **561**: p. 171-96.

5. Bryant, D.M., et al., *A molecular network for de novo generation of the apical surface and lumen.* Nat Cell Biol, 2010. **12**(11): p. 1035-45.
