# Supplementary figures and images for: PPAR-gamma induced AKT3 expression increases levels of mitochondrial biogenesis driving prostate cancer
Source: Oncogene. 2021 Mar 2;40(13):2355–66. doi: 10.1038/s41388-021-01707-7 (PMC8016665; doi:10.1038/s41388-021-01707-7)

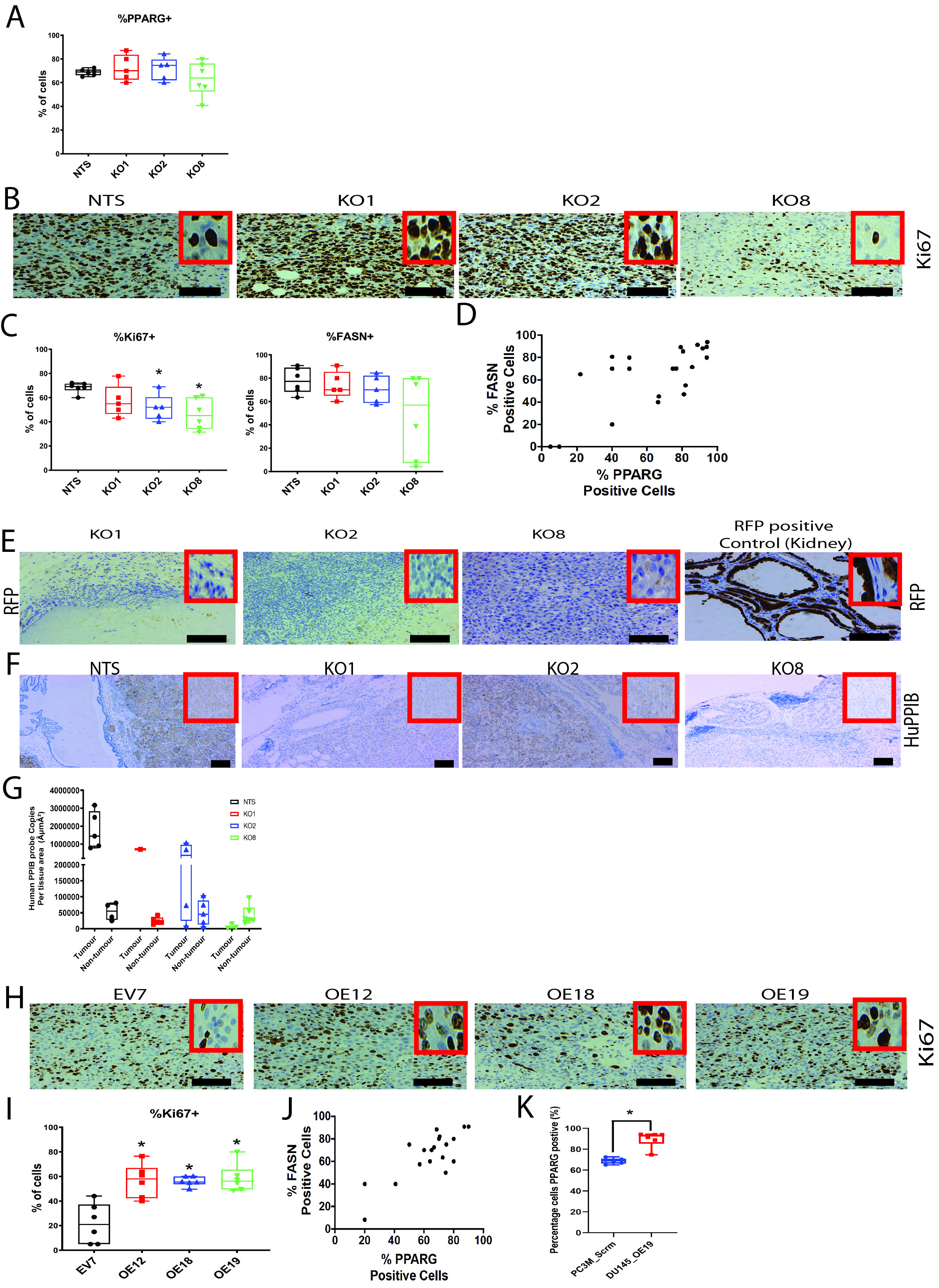

Supplement: Supplementary file 3 — Supplementary Figure 1 [file 41388_2021_1707_MOESM3_ESM.jpg]

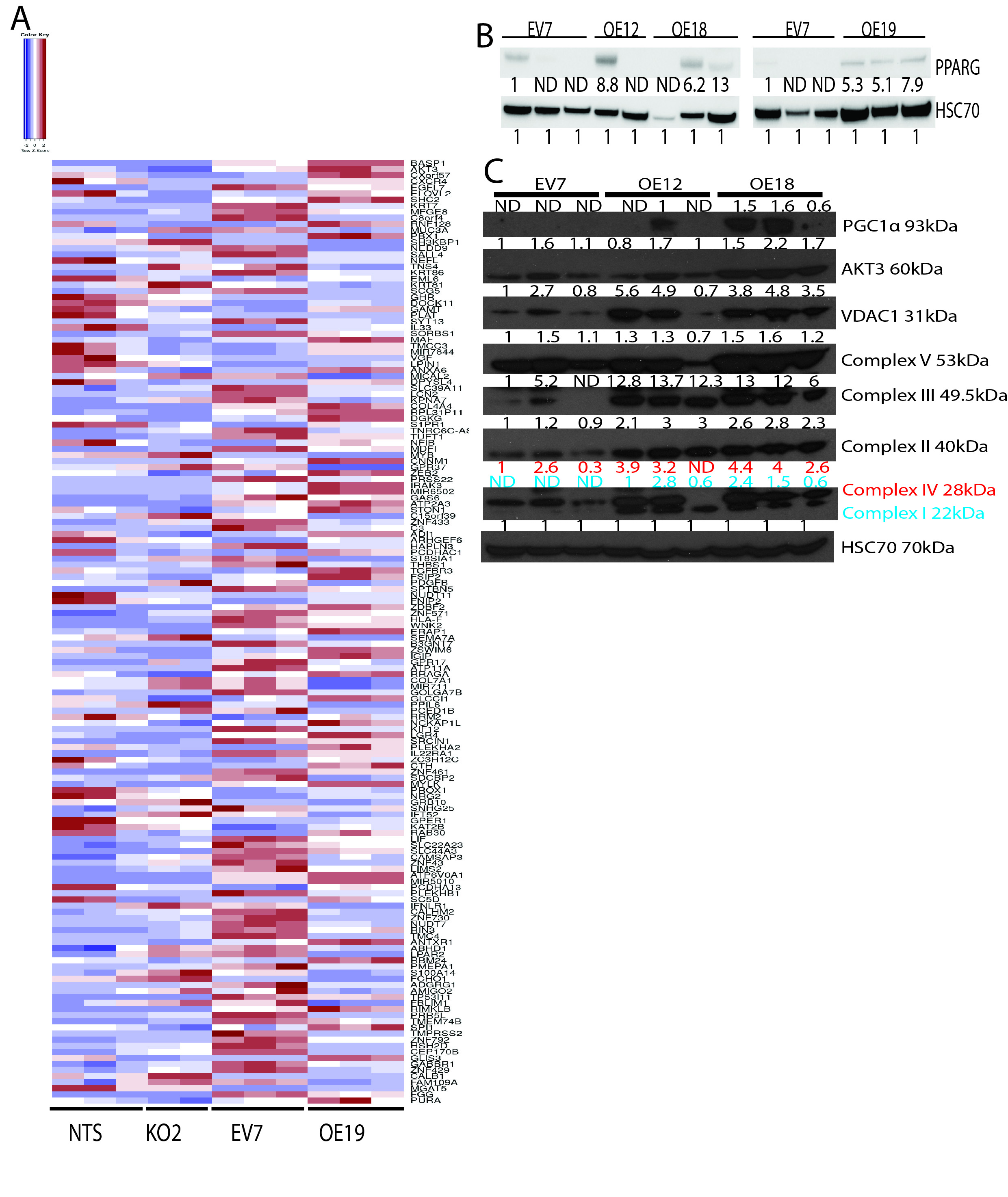

Supplement: Supplementary file 4 — Supplementary Figure 2 [file 41388_2021_1707_MOESM4_ESM.jpg]

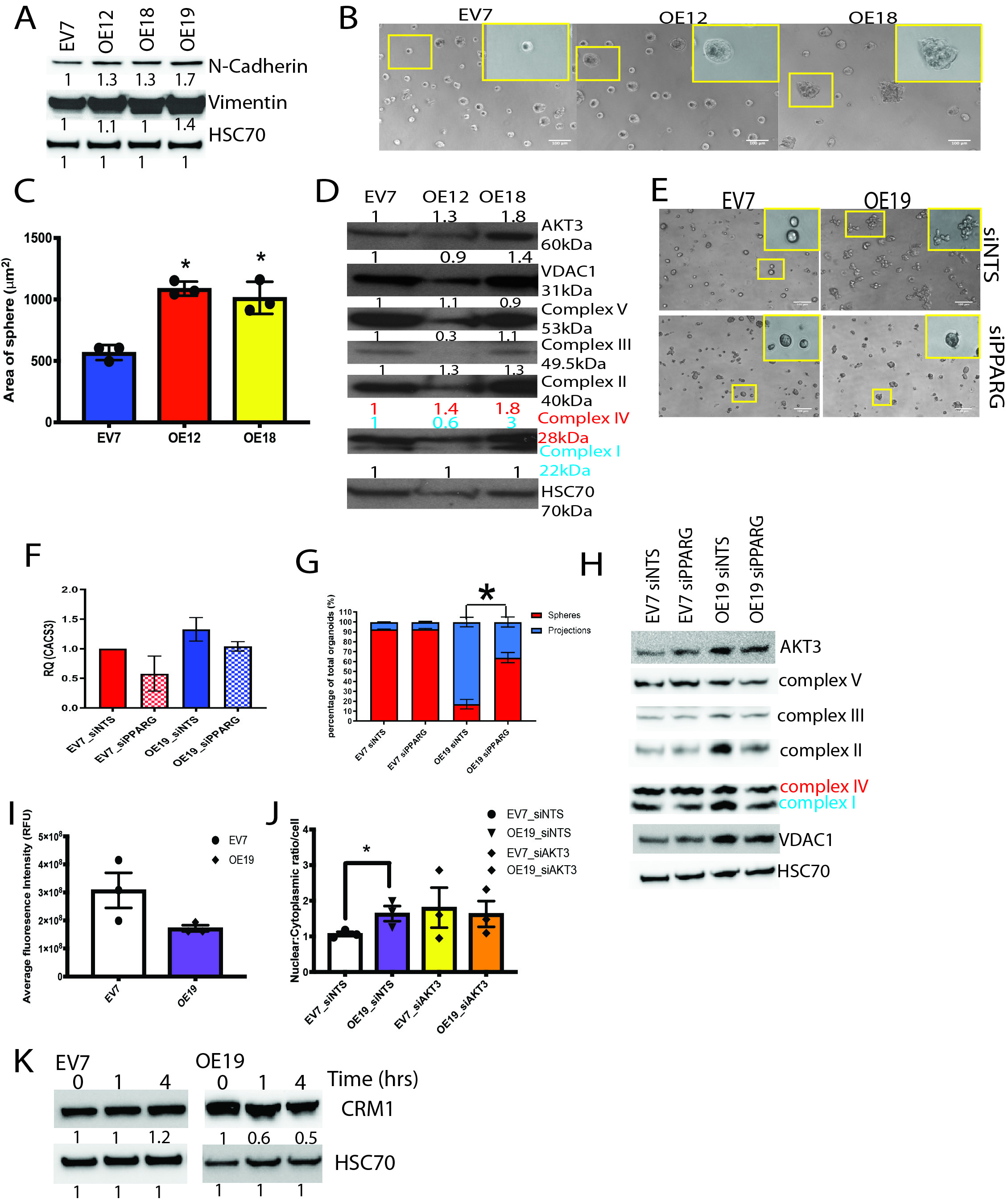

Supplement: Supplementary file 5 — Supplementary Figure 3 [file 41388_2021_1707_MOESM5_ESM.jpg]
